# Anatomic Variation in the Origin and Course of Radial Artery: A Descriptive Cross-Sectional Study

**DOI:** 10.31729/jnma.4609

**Published:** 2019-12-31

**Authors:** Muna Kadel, Shanta Hada, Bishwo Prachanda Sedhain

**Affiliations:** 1 Department of Anatomy, Nepalese Army Institute of Health Sciences, Sanobharyang, Kathmandu, Nepal; 2 Department of Anatomy, KIST Medical College and Teaching Hospital, Lalitpur, Nepal; 3 Samaj Dental Hospital, Baneshwor, Kathmandu, Nepal

**Keywords:** *anatomic variation*, *angiography*, *cadaver*, *radial artery*

## Abstract

**Introduction:**

The radial artery is commonly originated from the brachial artery in the cubital fossa at the level of the neck of the radius. It is the artery of choice for coronary artery angiography, percutaneous coronary artery intervention, cannulation, and others. Radial artery anomalies like high origin, tortuosity, and accessory branches are associated with the failure of such procedures. The main objective of this study is to study the variation in origin and course of the radial artery in cadavers.

**Methods:**

A descriptive cross-sectional study was conducted in 27 formalin-fixed adult human cadavers in the Department of Anatomy, KIST Medical College and Teaching Hospital, Lalitpur, Nepal, from 2075/4/2 to 2076/4/2. Ethical approval was taken on date 02/04/2075 (IRC No. 2074/75/38). Altogether, 53 specimens were enrolled in the study by convenience sampling method. Point estimate at 95% Confidence Interval was done for binary data along with frequency and proportion. Data were entered and calculationswere done by and Statistical Package for Social Sciences version 20.

**Results:**

Out of of 53 upper limbs, forty-six (86.79%) specimens, the origin of the radial artery was observed to be normal in the cubital fossa, 34.5±6.31mm below the level of the intercondylar line of the humerus with the superficial course. In seven (13.21%) specimens, the radial artery showed variation in the origin. Among them, variations in origin were found to be from sites like the axilla, upper-middle, and lower part of the arm. One cadaver showed a tortuous radial artery bilaterally.

**Conclusions:**

Most of the radial artery originates in the cubital fossa from the brachial artery with few variations.

## INTRODUCTION

Radial artery (RA) is commonly originated from the brachial artery in the cubital fossa at the level of the neck of the radius. Sometimes it can display higher origin from brachial or even from the axillary artery and termed as brachioradial artery. Proximally, it lies deep to the belly of brachioradialis, but distally it is covered only by the skin, superficial and deep fascia.^1^

RA is the artery of choice for coronary artery angiography, percutaneous coronary artery intervention, coronary artery bypass graft surgery, cannulation, and others.^2^ RA anomalies like high origin, tortuosity, accessory branches are associated with failure of transracial coronary artery procedures.^3^ When the superficial radial artery persists, it is more vulnerable to accidental injuries. It can be easily mistaken as a vein and intraarterial injections into it can be disastrous.^4^

The main objective is to study the variation in origin and course of the radial artery in cadavers.

## METHODS

A descriptive cross-sectional study was conducted in the Department of Anatomy, KIST Medical College and Teaching Hospital, Lalitpur, Nepal. After obtaining ethical approval from the Institutional Review Committee (IRC no. 2074/75/38), data was collected from 27 embalmed cadavers from 2075/4/2 to 2076/4/2. Properly dissected adult human cadavers of both sexes with intact blood vessels were included in the study. Cadavers with any limb anomalies and cut radial artery were excluded from the study. The fixed specimens (53 limbs) were partially dissected by the first year MBBS students during their routine dissection following the steps of Cunningham's dissection manual vol.1. The procedures were followed in accordance with the ethical standards of handling of a cadaver for teaching and learning purposes and further necessary dissection was done by the investigators.

Sample size was calculated by using the following formula,

$$\begin{array}{ll} \text{n} & ={\text{Z}}^{\text{2}}\times \text{(p}\times \text{q)}/{\text{e}}^{\text{2}}\\ & ={\text{(1.96)}}^{\text{2}}\times \text{0.908}\times \text{(1}-\text{0.908)}/{\text{(0.08)}}^{\text{2}}\\ & =50.1 \end{array}$$

where,

n= minimum required sample size
Z= 1.96 for 95% Confidence Interval
p= prevalence of the normal origination of radial artery from previous study as 90.8%*
e= margin of error, 8%
(*Hatadaj R et al. 2018)^1^

Altogether, 53 specimens were enrolled in the study by convenient sampling method. Specimens were numbered from 01 to 53. The radial artery was studied with respect to its origin and course.

The measurements were taken by digital Vernier caliper at an accuracy of 0.0001mm, which includes:

1. Origin level of the radial artery in relation to the interepicondylar line of the humerus,
2. Length of the radial artery,
3. Forearm length from head to the distal end of the styloid process of the radius.

Variations of the radial artery in cadavers were tagged, and photographs were taken. All the observations were recorded and tabulated. The data was analyzed with the help of SPSS version 20 software. The descriptive data analysis was done to find the mean and standard deviation of the level of origin of the radial artery, length of radial artery, and length of forearm of the cadavers.

## RESULTS

The variation in the origin of the radial artery was observed in 7 (13.21%) cases. In forty-six (86.79%) specimens of fifty-three upper limbs, the origin of the radial artery was observed to be normal, in the cubital fossa, below the level of the intercondylar line of the humerus.

**Figure 1:**
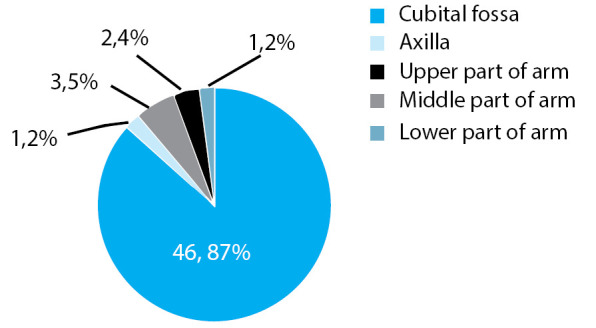
Pie chart showing site of origin of radial artery.

The following variations in the origin of the radial artery were observed. In one (1.88%) upper limb specimen, the radial artery was found to be originated from the 3^rd^ part of the axillary artery,

In three (5.66%) upper limb specimens, the radial artery was originated from the upper 1/3rd of the arm, and in two (3.77%) specimens, it was originated from the middle third of the arm as brachioradial artery. In one (1.88%) of the specimen, it was originated from the lower art of the arm (Figure 1).

The course of the brachioradial artery was superficial. The artery crossed the median nerve superficially from medial to the lateral side and descended on its lateral side down to the cubital fossa lateral to the tendon of biceps brachii. After that, it followed the normal course and distribution within the forearm and hand.

The mean distance between the intercondylar line of the humerus and the RA origin was 36.3mm. Similarly, the mean length of the RA was 23.69±1.63cm. The length of the left forearm is slightly greater than that of the right side. In addition, the mean length of the forearm from the head to the distal end of the styloid process of the radius was 23.99±1.48cm.

The mean of all three parameters related to the radial artery was determined on both sides (Table 1).

**Table 1:** Mean and SD of different parameters of radial artery of right and left side (n=53).

RA parameters	n	Minimum	Maximum	Mean±SD
RA origin level in relation to intercondylar line-right (mm)	27	26.98	50.00	38.0855±7.50778
RA origin level in relation to intercondylar line-left (mm)	26	27.32	47.78	34.5290±6.31373
length of RA-right (cm)	27	20.50	26.00	23.6600±1.59321
length of RA-left (cm)	26	20.00	26.30	23.6900±1.63350
Fore arm length-right (cm)	27	21.00	26.50	23.9950±1.48554
Forearm length-left (cm)	26	22.00	26.50	24.1632±1.28721

In one (1.88%) cadaver radial artery was found to be tortuous bilaterally.

## DISCUSSION

According to this study, in most cases, 46 (86.79%) the origin of the radial artery was found to be normal that is from the cubital fossa. In 7 (13.21%) cases, the radial artery showed variation in the origin like in the axilla from the axillary artery or the upper part of the brachial artery in the arm. The origin of the radial artery from the intercondylar line was found to be 36.3mm distally. Similarly, the length of the radial artery and forearm length was found to be 23.67cm and 24.07cm, respectively. Based on these results, it is concluded that most of the cadavers of KIST Medical College show the normal pattern of origin of the radial artery from the brachial artery in the cubital fossa 36.3mm below the intercondylar line of the humerus.

Similar to this study, the classical origin of the RA, as one of the two terminal branches of the brachial artery within the cubital fossa was seen in 92% to 94.8% of upper limbs in the study conducted by Nasrand.^5^ Radial artery arising from axillary artery was reported as 1.67% by Haladaj, et al. and 3.1% by Rodriguez-Niedenfuhr, et al.^1,6^ Similar variation was observed to be about 1.88% in this study. Variation in the origin of the radial artery has got an embryological background.^1^ In this study, 11.32% of cases radial artery was found to be originated from the upper part of the brachial artery as brachioradial artery. This finding was similar to the finding of Karlsson, et al. (8.54%) and Hassan, et al. (6.2%).^7,8^

In this study, the mean distance between the intercondylar line of the humerus and the normal origin of RA was 36.3mm, which was similar to the study conducted by Nasr, which is 36.5±8.5mm. According to the study of A.Y. Nasr, the mean length of the RA was 22.62±2.17cm which was similar to the finding of this study that is 23.66±1.59cm.^5^

In the study conducted by Hassan AK et al., 2.1% had extreme radial artery tortuosity.^7^ In this study also 1 (1.88%) cadaver showed bilateral tortuous radial artery.

Nowadays, radial access has emerged as the default strategy for both diagnostic and interventional procedures. However, anatomic variations at the level of the radial artery such as high radial artery origin, loops, and tortuosities, are not uncommon and can be associated with prolonged procedural duration or even can generate more procedural failures.^9^ For these reasons, it is suggested to do a preliminary angiogram of the arteries of the forearm.

## CONCLUSIONS

In this study, it is concluded that most of the radial artery originates in the cubital fossa from the brachial artery with few variations along with a tortuous radial artery.

## References

[1] Haladaj R, Wysiadecki G, Dudkiewicz Z, Polguj M, Topol M (2018). The High Origin of the Radial Artery (Brachioradial Artery): Its Anatomical Variations, Clinical Significance, and Contribution to the Blood Supply of the Hand. Biomed Res Int.

[2] Piers LH, Vink MA, Amoroso G (2016). Transradial Approach in Primary Percutaneous Coronary Intervention: Lessons from a High-volume Centre. Interv Cardiol.

[3] Sandhu K, Butler R, Nolan J (2017). Expert Opinion: Transradial Coronary Artery Procedures: Tips for Success. Interv Cardiol.

[4] Yang H, Gil Y, Lee H (2008). Variations of the Superficial Brachial Artery in Korean Cadavers. J Korean Med Sci.

[5] Nasr AY (2012). The radial artery and its variations: anatomical study and clinical implications. Folia Morphol.

[6] Rodriguez-Niedenfuhr M, Vazquez T, Nearn L, Ferreira B (2002). Variations of the arterial pattern in the upper limb revisited: a morphological and statistical study, with a review of the literature. J Anat.

[7] Karlsson S, Niechajev IA (1982). Arterial anatomy of the upper extremity. Acta Radiol Diagn.

[8] Hassan AKM, Alkhateeb T (2016). Radial artery anomalies in patients undergoing transradial coronary procedures-An Egyptian multicenter experience. Egypt Heart J.

[9] Valsecchi O, Vassileva A, Musumeci G, Rossini R, Tespili M, Guagliumi G (2006). Failure of transradial approach during coronary interventions: anatomic considerations. Catheter Cardiovas Interv.

